# Differential chemotactic receptor requirements for NK cell subset trafficking into bone marrow

**DOI:** 10.3389/fimmu.2013.00012

**Published:** 2013-01-30

**Authors:** Giovanni Bernardini, Giuseppe Sciumè, Angela Santoni

**Affiliations:** ^1^Laboratory of Immunology and Immunopathology, Department of Molecular Medicine and Istituto Pasteur-Fondazione Cenci Bolognetti, “Sapienza” Università di RomaRome, Italy; ^2^NeuromedPozzilli, Italy

**Keywords:** NK cells, chemokines, trafficking, bone marrow, egress

## Abstract

Responsiveness of maturing natural killer (NK) cells to chemotactic molecules directly affect their retention and relocation in selected bone marrow (BM) microenvironment during development, as well as their localization at sites of immune response during inflammatory diseases. BM is the main site of NK cell generation, providing microenvironmental signals required to sustain cell proliferation and differentiation. Drastic changes of expression and function of several chemoattractant receptors can be observed during progression from precursor NK cells to immature and mature NK cells. Indeed, the gradual decrease of CXCR4 expression parallels the increased expression of CXCR3, CCR1, and CX3CR1 and S1P_5_ (Sphingosine-1-phosphate receptor 5) on mature DX5^+^ NK cells. The chemokine CXCL12 is produced constitutively in the BM and, acting via CXCR4, is critical for retaining immature and mature NK cell subsets in the BM. During steady-state, the maintenance of NK cells into BM parenchyma depends on the equilibrium of CXCR4 retention and S1P_5_ mobilizing functions, as the gradient of S1P coming from the sinusoids facilitates mature NK cell egress into circulation via S1P_5_, when CXCR4/CXCL12-mediated retention decreases. Chemoattractants are also key factors for the response to inflammatory or infection conditions that promote mobilization of effector NK cells from storage compartments (including BM) to sites of disease or for NK cell recruitment/response during pathological conditions that affect BM integrity, including hematopoietic malignancies. In this review, we summarize what is known about the requirement for NK cell localization and exit from BM and how chemokine-mediated functions may affect BM NK cell development and immune responses.

Natural killer (NK) cells are innate lymphocytes that provide host protection against infection diseases and cancer through recognition of ligands on infected and transformed cells mediated by the combination of a number of activating and inhibitory receptors (Lanier, 2008). The final signaling outcome of these receptors is responsible for the effective control of virus infection and tumor initiation and dissemination, obtained through induction of NK cell cytokine production (i.e., IFN-γ), secretion of granules or expression of death inducing ligands of TNF superfamily receptors (i.e., TRAIL).

## NK CELL DEVELOPMENT IN THE BONE MARROW

Ablation of bone marrow (BM) hematopoiesis irreversibly alters NK cell development, whereas the absence of other lymphoid organs including the spleen or thymus through disease or removal does not result in reduced NK cell number, indicating a pivotal role of BM in NK cell development (Kumar et al., 1979; Seaman et al., 1979; Schwarz and Hiserodt, 1990; Ramos et al., 1996). Nevertheless, peripheral NK cells from several extramedullary tissues display tissue-specific phenotypes suggesting that maturation can be completed in organs different from BM including thymus, spleen, lymph nodes (LNs), and liver (Takeda et al., 2005; Hayakawa and Smyth, 2006; Vosshenrich et al., 2006; Münz, 2008).

Natural killer cells can be identified by expression of the activating NK receptors NK1.1 or NKp46 associated with the absence of T cell CD3 receptor complex. However, these cells are highly heterogeneous. In fact, during maturation, mouse NK cells gradually modulate the expression of several surface receptors. This process is driven by IL-15, and CD122 (β chain of IL-15/IL-2 receptor) expression is early acquired by the first committed NK cell precursor (pNK) and maintained by all NK cells (Rosmaraki et al., 2001; Kawamura et al., 2003; Colucci et al., 2003; Vosshenrich et al., 2005). CD127 (α chain of IL-7 receptor) is expressed only by a small percentage of immature NK cells (iNK) in the BM while IL-7 dependency has been shown for thymic-derived NK cells (Vosshenrich et al., 2006).

Integrin expression is tightly regulated by developing NK cells. In early stages of differentiation NK cells express low levels of CD11b (α_m_, also known as Mac-1) and high levels of CD51 (α_v_). Down-modulation of CD51 expression and acquisition of CD49b (α_2_, recognized by DX5 monoclonal antibody) marks the transition from an immature to a mature NK cell (Kim et al., 2002). Then, NK cells further mature by up-regulating CD11b expression, and subsequent stages of development are characterized by down-modulation of the tumor necrosis factor receptor superfamily member CD27 and expression of the killer lectin-like receptor G1 (KLRG1). The latter population is characterized by decreased ability to lyse target cells and produce IFN-γ as well as acquisition of a replicative senescence phenotype (Robbins et al., 2002; Hayakawa and Smyth, 2006; Huntington et al., 2007).

During differentiation, NK cells modulate in a coordinate manner the expression of several receptors for chemotactic factors (Bernardini et al., 2012). The chemokine receptor CXCR4 is expressed at the higher levels by pNK cells but its expression progressively decreases during development. The progressive decrease of CXCR4 parallels the increased expression of CXCR3, CCR1, and CX3CR1 on mature DX5^+^ NK cells, being the expression of CX3CR1 mainly acquired by the KLRG1^+^ subset (Sciumè et al., 2011). CX3CR1 expression is associated with lower expression of CXCR3, CCR1, and CXCR4. Later stages of NK cell maturation are also accompanied with the acquisition of the Sphingosine-1-phosphate receptor 5, S1P_5_ by the CD11b^high^CD27^-^ subset (Walzer et al., 2007).

## NK CELL SUBSETS AND TISSUE LOCALIZATION

Mature NK cells predominantly circulate in the peripheral blood, but are also resident in several lymphoid and non-lymphoid organs, such as spleen, tonsils, LNs, liver, lungs, intestine, and uterus (Bernardini et al., 2012). In all these organs, NK cells have been found in close proximity of vasculature, and in most instances in areas distinct from those of T or B cells. Different subsets of NK cells displaying distinctive functional features have been found in tissues, including thymus and liver, suggesting that they play specific roles during the induction of an immune response (Takeda et al., 2005; Vosshenrich et al., 2006; Hayakawa and Smyth, 2006; Münz, 2008; Paust et al., 2010).

In humans, the predominant NK cell population found in the LNs and tonsils is CD56^bright^ while the majority of peripheral blood NK cells are CD56^dim^ (Caligiuri, 2003). CD56^bright^ and CD56^dim^ NK cell populations are quite different, in that they have a distinct set of inhibitory and activating receptors and display diversity in their adhesion and chemokine receptor profile; thus they have different homing capability, and most importantly, they are endowed with unique functional ability being CD56^bright^ the major source of cytokines and the CD56^dim^ the major cytotoxic population (Cooper et al., 2001; Caligiuri, 2008).

Several mouse NK cell subsets displaying distinct functional capacities and tissue localization, can be identified also in the mouse when CD27 or KLRG1 are associated with CD11b (Hayakawa and Smyth, 2006; Huntington et al., 2007). On the other hand, CD56 is not expressed in rodent NK cells making it hard to find mouse counterpart of the CD56^bright^ and the CD56^dim^ NK cells. Functional characteristics similar to human CD56^bright^ NK cells were found in the mouse thymic NK cells that can be distinguished by their expression of CD127 and GATA3 (Vosshenrich et al., 2006). CD127^+^ NK cells are dependent on IL-7 and the transcription factor Gata-3, lack CD16 expression and most of Ly49 receptors, preferentially home to LNs, and are biased toward cytokine production with reduced cytotoxic capacity.

In the liver, NK cells are preferentially located in the hepatic sinusoids, often adhering to the endothelial cells (Doherty and O’Farrelly, 2000). In mouse, these cells are mainly CD11b^low^CD27^+^ and express the TNF-related apoptosis-inducing ligand (Trail), while they lack expression of CD49b and most Ly49 receptors as well as perforin and granzymes (Hayakawa and Smyth, 2006). These cells specifically require the transcription factor T-bet for their development (Gordon et al., 2012). A discrete subset of CXCR6^+^ NK cells located in the liver sinusoids acquires and retains antigen-specific memory of viral antigen. CXCR6^+^ NK cell maintenance in the liver is dependent on this chemokine receptor, which regulates the effector function and survival of memory NK cells. Finally, liver NK cells have also been described as a major source of IL-10 producing cells, and a role of sinusoidal NK cells in the elimination of hepatocytes during infection and of stellate cells during resolution of liver fibrosis has been shown (Lee et al., 2009; Perona-Wright et al., 2009; Paust et al., 2010).

## ROLE OF CHEMOTACTIC FACTORS IN NK CELL MIGRATION IN AND OUT THE BM DURING STEADY-STATE

Mechanisms operate under basal conditions to maintain NK cell numbers in tissues. Among them, directed migration is essential at several stages of the NK cell life cycle including: (1) precursor movement toward the sinusoids in the BM; (2) migration of distinct NK cell population through the sinusoidal endothelium into systemic circulation; (3) recruitment into tissues.

Several chemoattractant molecules exposed on the surface of endothelial cells recruit lymphocytes by acting on G protein coupled receptors (GPCRs) that activate integrins causing adhesion to complementary molecules on the venular endothelium. Among chemoattractants, chemokines are small cytokines with pleiotropic functions having effect on a broad range of leukocytes (Bonecchi et al., 2009). So far, more than 50 ligands and 20 receptors have been described in humans and mice. Based on the presence of conserved cysteine residues there are two major (CXC and CC) and two minor (C and CX3C) chemokine classes and accordingly four classes of chemokine receptors (CXCR, CCR, CX3CR, XCR). Another chemoattractant, the lipid S1P and its receptors (S1P_1__-__5_) are required for lymphocyte egress from lymphoid organs. S1P is synthesized by most cells, but then is irreversibly degraded by intracellular S1P lyase or dephosphorylated by S1P phosphatases. Thus, S1P levels are extremely low in tissues while they are maintained elevated in the blood and lymph, allowing the formation of a gradient of S1P that maintains its concentration elevated at tissue exit site (Cyster and Schwab, 2012).

**FIGURE 1:**
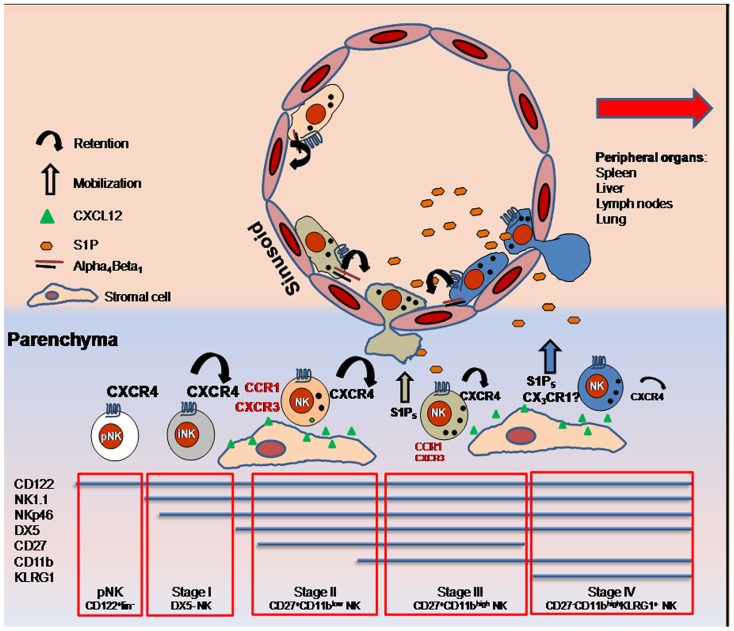
**Expression of several chemoattractant receptors is modulated on NK cells maturing in BM.** During steady-state, the maintenance of NK cells into BM parenchyma depends on the equilibrium of CXCR4 and S1P_5_ function, as the gradient of S1P coming from the sinusoids facilitates mature NK cell egress into circulation via S1P_5_, while CXCR4/XCL12-mediated retention decreases. Prevalent CX3CR1 expression by KLRG1^+^ NK cells located in sinusoids is suggestive of a role of this receptor in NK cell entry into the vascular compartment. NK cells are maintained into sinusoids through alpha4 integrin-dependent mechanisms. Inflammatory receptors (written in red) are also up-regulated during maturation and regulate NK cell trafficking into BM during inflammation. Size of the alphabetic characters indicate level of receptor expression.

Natural killer cell egress from lymphoid organs is necessary for immune surveillance and for effector cell trafficking to sites of inflammation. Under homeostatic conditions, trafficking of NK cells into BM is mainly governed by the opposite role played by the chemoattractant receptors CXCR4 and S1P_5_. CXCR4 is highly expressed by pNK cells and iNK cells. Successively its expression decreases in parallel with NK cell maturation (Bernardini et al., 2008). This chemokine receptor is important to retain NK cells in the BM parenchyma as shown by promotion of parenchymal NK cell mobilization following *in vivo* administration of the CXCR4 pharmacological antagonist AMD3100 (Sciumè et al., 2011). In the BM, CXCL12 is expressed by osteoblasts located in the endosteal region, and CXCL12-abundant reticular (CAR) cells, which are uniformly distributed throughout the BM (Petit et al., 2002; Tokoyoda et al., 2004). BM NK cells are found in proximity to CAR cells that include a fraction of cells able to express IL-15 together with IL-15Rα and thus might support NK cell development (Noda et al., 2011). It was previously demonstrated that KLRG1^+^ NK cells, also defined as CD11b^high^CD27^low^, have a markedly reduced CXCR4-requirement for retention in BM. Indeed, reduction of CXCR4 retention activity and the concomitant engagement of S1P_5_ (expressed by CD11b^high^CD27^low^ cells; Walzer et al., 2007), allows NK cells to leave the parenchyma and to move to the blood through the sinusoids. Although CXCR4 desensitization is S1P_5_ independent, both the release of CXCR4-mediated retention and activation of S1P5 are necessary for NK cells to reach the sinusoids (Mayol et al., 2011). Ten to twenty percent of the total BM NK cells reside in this compartment, and their localization in this site is mainly dependent on the integrin chain α_4_. Indeed, the treatment *in vivo* of C57BL/6 mice with a specific anti-α_4_ blocking antibody is able to mobilize all the sinusoidal NK cells to the periphery (Sciumè et al., 2011). In addition, about 80% of KLRG1^+^CX3CR1^+^ BM NK cells are located in sinusoids, suggestive for a role of this receptor in sinusoidal NK cell localization or in NK cell exit from BM parenchyma under steady-state. Of note, the specific ligand for CX3CR1, the chemokine CX3CL1/fractalkine, was also shown to be expressed by human BM cells, although the distribution pattern (i.e., vascular versus parenchymal) of the chemokine has not been clearly defined (Jamieson et al., 2008). Thus, multiple chemoattractant receptors play a role (mobilization versus retention) in the regulation of NK cell egress from the BM (**Figure 1**). Whether these receptors are co-expressed or are expressed on different NK cell subsets is an important issue to be addressed in order to better define their relative impact on the maintenance of NK cell populations in BM.

## ROLE OF CHEMOTACTIC FACTORS IN NK CELL MIGRATION IN AND OUT THE BM DURING PATHOLOGICAL CONDITIONS

Beside CXCR4, other two CXC chemokine receptors, CXCR3 and CXCR6, are expressed by NK cells, but their role in NK cell trafficking into BM during steady-state is unclear. Indeed, CXCR3 seems to play a major role in regulating NK cell trafficking outside the BM during inflammatory conditions (Beider et al., 2003; Wald et al., 2006; Gregoire et al., 2007). Beider et al. (2003) analyzed the homing of unstimulated or IL-2-treated human NK cells after transfer into immunodeficient mice. While CXCR4 expression promoted homing of unstimulated NK cells to BM, down-regulation of CXCR4 and up-regulation of CXCR3 obtained following *in vitro* stimulation with IL-2 inhibited their migration in response to CXCL12 and their homing and retention in the BM. In agreement with a prevalent role of CXCR4 in NK cell BM tropism during homeostasis, NK cell trafficking to BM was observed only when non-irradiated immunodeficient animals where used in a model of allogeneic BM transplantation (Olson et al., 2009). Conversely, donor NK cell accumulation in the BM was selectively impaired in conditioned mice, indicating that NK cell trafficking and expansion in BM may be driven by homeostatic mechanisms that are negatively affected by inflammation.

Bone marrow is a storage compartment for mature NK cells to be mobilized in inflammatory conditions. Indeed, mobilization of NK cells from BM markedly contributes to their recruitment into the liver in mouse models of hepatic inflammation promoted by Concanavalin A as well as by mouse cytomegalovirus (MCMV) infection (Salazar-Mather et al., 1996; Wald et al., 2006). Previous studies have shown that CCL3, the ligand for the chemokine receptors CCR1 and CCR5, plays a crucial role in the recruitment of BM-derived NK cells to the inflamed liver during MCMV infection (Salazar-Mather et al., 1996). The increased plasma levels of CCL3 that occur during liver infection can also directly enhance NK cell exit from BM to the periphery, as shown by *in vivo* administration of the recombinant chemokine CCL3 (Bernardini et al., 2008). Interestingly, CCL3-promoted mobilization was facilitated by CCL3-induced down-modulation of CXCR4 expression and function. Thus, while during homeostatic conditions, desensitization of CXCR4 occurs independently of mobilizing stimuli (i.e., S1P), chemokines released during inflammation can favor NK cell entry into circulation by acting both directly on the migration of BM NK cells and, indirectly, by reducing their CXCR4-dependent retention control.

Beside egress of effector NK cells, a selective migration into BM of CD11b^+^CD27^-^ long-lived NK cells was also described during influenza virus infection (van Helden et al., 2012). This population was maintained into BM, underwent homeostatic proliferation and was able to proliferate in response to a new infection. Since BM was the only site where proliferation was observed during infection, it has been hypothesized that this population is important to support NK cell accumulation at infection site and constitute a reservoir of NK cells able to rapidly expand in response to a new infection.

CXCL12 and other chemokines are produced by BM cells under physiological conditions. Bone marrow stromal cells (BMSC) isolated from subchondral bone during steady-state secreted a ligand for CXCR1 and CXCR2, CXCL8, and a ligand for CCR2, CCL2 (Lisignoli et al., 1999). While the relevance of these chemokines under steady-state conditions is still unclear, KC, another ligand for the mouse CXCR2, and CCL2 were shown to play a critical role in the egress from BM into blood of leukocyte populations during activating conditions, i.e., G-CSF treatment or infection, respectively (Martin et al., 2003; Serbina and Pamer, 2006). Interestingly, this effect was associated with their enhanced expression in proximity to the vascular compartment, thus facilitating target leukocyte migration toward blood circulation (Köhler et al., 2011; Shi et al., 2011).

In human joint diseases, such as osteoarthritis and rheumatoid arthritis, NK cells were shown to constitute a large fraction of synovial joint infiltrate and to support osteoclastogenesis thanks to their expression of the receptor activator of NF-κB ligand (RANKL; Huss et al., 2010; Söderström et al., 2010). Beside expression of chemokines in the inflamed joints, significantly higher levels of CXCL8, CXCL1, and CCL5 are produced by BMSC during disease and may contribute to recruitment of effector cells into BM parenchyma or to their egress into circulation (Lisignoli et al., 1999; Haringman et al., 2006; Huss et al., 2010). Nevertheless, NK cell distribution within the immune cell infiltrate of subchondral bone in inflammatory joint disease has not been investigated yet.

The BM is the site of disease of most hematological tumors, and increasing evidence demonstrates that the endogenous immune response toward the malignant cells has a critical role in preventing tumor progression. When tumor progress, hematopoietic stem cell transplantation (HSCT) is considered a promising therapeutic strategy for the cure of hematologic malignancies for patients lacking an HLA-identical donor. NK cells mediate anti-tumor activity without causing graft versus host disease (GVHD) and are thus under evaluation for cellular immunotherapy after haploidentical HSCT (Ruggeri et al., 1999; Farag et al., 2002; Frohn et al., 2002). Although NK cell anti-tumor activity *in vivo* is likely dependent on the recruitment of NK cells to the tumor site, the mechanism of NK cell homing to BM following adoptive immunotherapy is still unclear. In a mouse model of multiple myeloma (MM), tumor clearance was associated to effector cell homing to tissues infiltrated by MM cells, including BM, when IL-2-activated NK cells were adoptively transferred to MM-bearing mice (Alici et al., 2007). In addition, during MM disease progression (Han et al., 2001; Giuliani et al., 2006), expression of several chemokines potentially capable of promoting NK cell migration *in vivo* was shown to be up-regulated in BM.

Besides their role in limiting primary tumor growth in BM, NK cells were also shown to prevent bone metastasis of a number of solid tumors (Lode et al., 1998; Smyth et al., 1999; Bidwell et al., 2012). In this context, the importance of NK cell homing to BM is unclear as the contribution of NK cells against *in situ* metastatic tumor cells with respect to circulating tumor cells is still poorly investigated.

## CONCLUDING REMARKS

Beside a prominent function in development, correct localization of NK cells in BM have a fundamental role in several aspects of NK cell-mediated immune response *in vivo*. Thus, the study of chemoattractant-mediated NK cell trafficking to BM and their regulation of NK cell migration in selected BM niches is directly linked to the understanding of the mechanisms of NK cell function *in vivo*. Chemoattractants are key factors for the response to inflammatory or infection conditions that promote mobilization of effector cells from storage compartments (including BM) to sites of disease or for NK cell recruitment/response during pathological conditions that affect BM integrity, including hematopoietic malignancies. Additionally, recent observations indicate that long-lived NK cells undergo homeostatic proliferation into BM during viral infections. This is reminiscent of what happen to memory CD8 T cells that proliferate more extensively in the BM than they do in peripheral organs (Parretta et al., 2005) and suggests that activated NK cell homing to BM is needed to maintain a population of cells more responsive to previously experienced pathogens. In these contexts, it will be important to address which chemoattractants can determine the specificity of niche–NK cell interaction, and to identify molecular mechanisms by which BM cells regulate generation, maintenance, and exit of NK cells during homeostasis, microbial infection, inflammation, and hematological malignancies.

## Conflict of Interest Statement

The authors declare that the research was conducted in the absence of any commercial or financial relationships that could be construed as a potential conflict of interest.
